# The effectiveness of oral antiviral (Sofosbuvir/Ledipasvir) in treating children with HCV infection

**DOI:** 10.12669/pjms.346.15722

**Published:** 2018

**Authors:** Ban Adil Alkaaby, Abd El-Salam Al-Ethawi

**Affiliations:** 1 *Ban Adil Al-Kaaby, MRCPCh, FICMS-Ped., Senior Lecturer, Paediatrics Department, Al-Mustansiryah Medical College, Specialist Pediatrician at Central Child Teaching Hospital & Gastroenterology and Hepatology Center, Baghdad, Iraq*; 2 *Abd El-Salam Al-Ethawi, Senior Lecturer, Pediatrics Department, Al-Mustansiryah Medical College, Paediatrics Cardiologist at Ibn Al-Bittar Center for Cardiac Surgery and Central Child Teaching Hospital, Baghdad, Iraq*

**Keywords:** Direct acting antivirals, Hepatitis C virus, Sofosbuvir/ledipasvir, Pediatrics, Children

## Abstract

**Objectives:**

To determine the efficacy of (Sofosbuvir/ledipasvir) in treating children with HCV infection.

**Methods:**

This study was conducted at Gastroenterology, Hepatology Center /Pediatrics department and the Central Child Teaching Hospital, Baghdad / Iraq from April 2017 to January 2018. Patients with positive HCV PCR, aged 7 to 18 years were enrolled. History, clinical examinations and investigations were conducted. HCV genotyping was done (if affordable). Sofosbuvir/Ledipasvir was given to all patients once daily. Ribavirin was added for INF-experienced patients or with established cirrhosis. Follow up with liver function and renal function and PCR was done at 12 weeks (end of treatment); then after 12 weeks post treatment (SVR12). Total duration of therapy was 12 weeks, extended to 24 in cases with established cirrhosis. Computer program SPSS version 20 was used for data analysis.

**Results:**

The number of patients was 22, with mean age of 12.5 years, 14 boys (63.6%), and 8 girls (36.4%). Genotype 1 was the dominant type (75%). SVR 12 was achieved in 20 patients (90.9%), the remaining two (9.1%) had partial virological response. HBV co-infection was found in five cases; they were kept on Entecavir during the course of treatment. All achieved SVR12 for HCV with decrease in titer of HBV. Even INF-experienced patients (7 patients 31.8%) were responsive with SVR12. The treatment was well tolerated.

**Conclusion:**

Sofosbuvir/ledipasvir is effective in treating HCV in children, and is well tolerated.

## INTRODUCTION

HCV has a major health impact worldwide. It is estimated according to the World Health Organization (WHO) latest report in 2015, that 71 million individuals are infected with HCV world widely. Regarding paediatrics, no data was provided in the WHO report yet a recent systematic review, estimated that the prevalence of HCV antibody in children was 13.2 (11.5–21.2) million children.^1^

The HCV treatment has evolved substantially after the approval of the first direct acting antiviral drug in 2011.^2^ More than 10 regimens have been licensed for treating HCV in adults. Each of these regimens can achieve sustain virological response (SVR) >90% with only 12 weeks oral treatment. Now treating chronic HCV in adults becomes much easier.^2^

Regarding the Pediatrics population, till very recent the only available regimen was the interferon plus ribavirin regimen. It’s SVR was less than required, as for genotype 1 and 4 the SVR reached to 64% in treatment-naïve patients, but may be even less reaching to only 50% when the viral load was high. While in genotype 2 and 3 the SVR was 89%.^3^

Other challenges that the interferon based regimen needed longer duration of treatment 24-48 weeks. This also needed close follow up. A long list of side effects was well known with the Interferon and ribavirin that causes poor compliance or necessitate dose modification.^3^,^4^

In April 2017, the FDA had finally approved the use of Sofosbuvir/Ledipasvir in adolescence, more than 12 years,^5^ further studies evaluated its use for children 6-11ys with high efficacy, and safety.^6^

In view of the above, we decided to use this treatment and to monitor its effectiveness in treating children more than six years with HCV. The aim of the study was to evaluate how effective and safe HARVONI (Sofosbuvir/Ledipasvir) was in treating Hepatitis C infection in children above six years of age.

## METHODS

HCV PCR positive patients referred to the Gastroenterology, Hepatology Center /Pediatrics department and the Central Child Teaching hospital, Baghdad / Iraq from April 2017 to January 2018 were interviewed. History, clinical examination and investigations were done for all enrolled patients. Complete blood count, total serum bilirubin, ALT, AST, alkaline phosphatase, abdominal ultrasound and HCV genotyping (if financially affordable by the patient).

All patients were started on Sofosbuvir 400 mg/ledipasvir 90mg once daily. If patients were less than 12 years, half the dose was given.^6^ Ribavirin 10-15 mg/kg/day was added if patient was INF-experienced or had established cirrhosis.^7^

All the patients were followed with liver function and renal function test in the 4^th^ week of treatment. PCR was done at 12 weeks (end of treatment); then after 12 weeks post treatment (SVR12). Total duration of therapy was 12 weeks. Extended to 24 in cases with establish cirrhosis. Computer program SPSS version 20 was used for data analysis.

## RESULTS

A total of 22 patients were included in this study, the mean age was 12.5 years the youngest patient received treatment was seven years old. There were 14 boys (63.6%), and 8 girls (36.4%). Genotype of the HCV was done by 12 (54.5%) patients only due to financial causes. Nine of them were genotype 1(75%) all were genotype 1a except one which was 1b. The remaining three cases (25%) were genotype 4. PCR level was high (more than one 800,000 IU/ml)^8^ in 14 patients (63.6%), mean level 9,016,862 IU/ml, minimum level 4,871 IU/ml and maximum level 35,175,925 IU/ml. SGPT was elevated in 9 cases (40.9%).

Cases referred from the Hematology and Oncology ward, with history of blood transfusion were 20 cases (86.36%) (thalassemia, leukemia, Hodgkin’s disease), one had a history of surgery, the other one had unknown source of infection.

Seven patients (31.8%) had previously been treated with Interferon; four were non-responsive, while one could not tolerate the side effects namely leukopenia and thrombocytopenia. The last two patients had developed autoimmune hepatitis during the course of treatment, for this reasons they were discontinued from the interferon regimen. Almost all apart from one of the treatment-experienced patients were genotype 1 (86%), genotype 4 only one case (14%). Concomitant infection with HBV was found in 5 patients, they were concomitantly treated with Entecavir or Tenofovir during the course of treatment.

Two cases from the sample had established hepatic cirrhosis, both had previous history of ALL (acute lymphoblastic leukemia), both of them had autoimmune hepatitis associated with the HCV, one had received interferon for few months but stopped because of no response.

End-of-treatment PCR was negative in 20 patients (90.9%), the remaining 2 (9.1%) had partial virological response. Those who had associated autoimmune hepatitis with established cirrhosis were responsive with negative PCR after 24 weeks of treatment. Those with concomitant HBV infection, they responded well and all had achieved negative PCV at the end of treatment. Those who were previously treated with INF, all achieved a negative HCV PCR at 12 weeks post treatment. The treatment was well tolerated, one patient only complaint from headache. It was due to sinusitis that was treated and there was no treatment interruption.

## DISCUSSION

Hepatitis C virus (HCV) is one of the major health problems world widely^9^ in spite of the advances in treating HCV in adult, data in paediatrics age groups are still limited.

The new oral antiviral drugs have revolutionized the treatment of HCV all over the word. Since FDA approved the use of (Ledipasvir and Sofosbuvir) in paediatrics age in April 2017, a new era in treating children with this disease has started.^5^ In this study, the youngest patient involved was six years, while another study in Pakistan had used it from five years old and above.^4^ The mean age of patients was 12.5 years. Male outnumbered female, the ration was about 2:1, same as in other study conducted in USA, UK, Canada.^6^

In this study the mode of acquiring HCV was from blood or blood product transfusion in most of the cases. Contemporary literature shows that the vertical transmission is considered the main source of infection in the developed countries due to effective screening of donor blood that reduced the blood born infection, in USA for instance no more pediatric cases acquired HCV from blood born since 1994.^10^ These findings are different from the developing countries where still the main source of infection blood born. 4,11-13 Adding to that HCV was noticed to be significantly higher among patients who received repeated blood transfusion; about 67.3% in Iraq, 40% in Saudi Arabia, 40.7% in Jordan, and 42.4% in Morocco.^13^ and almost all of our patients were referred from the hematological ward with history of blood and blood product transfusion.

In regard to HCV genotype only half of the cases were able to do the genotyping of the virus (54.5%) due to financial reasons. Genotype 1 was the dominant one followed by the Genotype 4, no case was genotype 2 nor 3. Previous study conducted in Iraq showed that in the south of Iraq 50% of their sample had Genotype 1 while 35% were genotype 4.^13^ While another study from the north of Iraq in patients with hemoglobinopathies found that 53% were Type-4, while Type-1 was 23% and 20.9% for Type-3 finally Type-2 in 2.3%.^14^ A systematic review and meta-analysis assessing the prevalence of HCV in the Arabian gulf region in 2016 shows that genotype 4 appears to be the dominant type in most of the region’s countries, then genotype 1 followed by genotype 3. This review supported that genotype 4 is also dominant in Egypt and in some MENA countries like Iraq, Jordan, Lebanon and Syria. Meanwhile, it shows that India, Pakistan and Nepal have genotype 1 and 3 more dominant than the others.^15^ This discrepancy may be attributed to that our study was not conducted on national base, as there is variability in the prevalence of the genotype between the country sites.

Patients who had been previously treated with INF were almost third of cases; 86% of them were genotype 1. This goes with previous studies which demonstrate that genotype 1 is more difficult to be treated with the PEG-IFN alpha-2b and Ribavirin regimen.^16^ it has a SVR 64% in naive patients, and decline to 50% if patient have high viral load. Unlike genotype 2 and 3 where SVR reach up to 85%.^3^,^16^ In spite of being genotype 1 and not treatment naïve patients, all the cases with these criteria reached SVR at 12 weeks. This is considered an evolution in HCV treatment, as even Interferon resistant cases had reached SVR12 Data about the use of these modality of treatment in pediatric are very limited, but a study shows that among 88 children using LDV/SOF plus ribavirin, 20% were treatment -experienced cases,99% of the cases shows SVR12 posttreatment.^6^ Co-infection with HBV is considered a risk factor for earlier development of complications as cirrhosis or hepatocellular carcinoma.^2^ Adding to that treatment with oral antiviral medication may cause flare up of HBV,^7^,^17^,^18^ hence all patient with HBV received anti HBV treatment(Entecavir or Tenofovir) during the course of treatment.

All reached to SVR12 for HCV and HBV PCR titter at the end of treatment decreased dramatically with no flare up recorded. This finding was comparable with the results of a study performed in Taiwan were there was 100% of the 111 patients who were HCV co-infected with HBV reached a SVR 12.^19^

The Extent of Viremia is a factor related to the response to oral antiviral.^20^ In this study, even those with high viremia, more than 800,000 IU/ml, were able to achieve a SVR, the two cases who did not achieve full viral response, did not have the higher PCR among the sample studies, yet larger sample needed to be studied, to evaluate the effectiveness in relation to viral load. Although many studies find that the lower viral load predict a better response with the INF- containing treatment, few were correlated with the oral antiviral.^21^,^22^

The treatment was well tolerated. Only one case reported to have headache, which is a reported side effect, but in this case after further investigations showed to have sinusitis which was treated, and this did not interfere with patient compliance. Other studies have reported other minor side effects as abdominal pain, headache, diarrhea, vomiting, nausea, fatigue, pyrexia, cough, and oropharyngeal pain. No patient needed to discontinue the treatment because of these side effects.^6^

Finally, what is most promising in this study is that SVR was achieved in 90.9%, which is considered a jump in the response compared to the old standard treatment were SVR was achieved in 50-64% only.^23^ This was comparable with two other studies performed on pediatric age, both shows promising results with 97.14%, 99% reaching SVR 12.^4^,^6^ The treatment was effective even in patients with risk factors as coinfection with HBV, or being previously treated for HCV with INF based regimen.

HCV was associated with AIH in 2 cases, they were on immune suppressive therapy and had established cirrhosis in spite of all the risk factors they had, and they responded and achieved SVR but needed longer duration of therapy about 24 weeks. Same comparable results were achieved in adults with the 24 weeks regimen in patients with cirrhosis and treatment experienced.^24^ These are promising results that can help in eradicating HCV from the paediatrics field as it has started in the adult field.

### Limitations of the study

The sample size was small due to the newly evolving regimen. The genotyping was unable to be done for all the cases because of the financial cost as is not funded by any governmental institute.

## CONCLUSION

Ledipasvir/sofospovir represents a highly effective and safe modality of treating HCV in Pediatrics. Further studies are needed to establish its use for younger age groups 3-6 years..
